# Fertility Prospects Among Testicular Cancer Survivors: Outcomes of Surgical Sperm Retrieval in Azoospermic Patients

**DOI:** 10.7759/cureus.96520

**Published:** 2025-11-10

**Authors:** Hatsuki Hibi, Miho Sugie, Yoshimasa Asada

**Affiliations:** 1 Urology, Kyoritsu General Hospital, Nagoya, JPN; 2 Urology, Dokkyo Saitama Medical Center, Koshigaya, JPN; 3 Obstetrics and Gynecology, Asada Institute for Reproductive Medicine, Nagoya, JPN

**Keywords:** intracytoplasmic sperm injection (icsi), microscopic sperm aspiration (mesa), sperm retrieval surgery, testicular cancer survivor, testicular sperm extraction (tese)

## Abstract

Introduction

Since general improvements in the prognosis of cancer patients have been achieved through advances in surgery, radiotherapy, and chemotherapy, subsequent infertility in adolescents and young adults (AYAs) has become a serious problem. Sperm quality is often abnormal at the time of testicular cancer diagnosis. Anti-cancer therapy further impairs spermatogenesis and, following orchidectomy, leaves patients with a single testis, raising long-term concerns about fertility. We therefore evaluated fertility outcomes among testicular cancer survivors attending our male infertility clinic.

Materials and methods

From 2004 to 2023, 3,361 male infertility patients consulted our division, 117 (3.5%) of whom had a history of cancer treatment. Thirty-nine patients diagnosed with testicular cancer were evaluated for cancer treatment, semen findings, and infertility treatment. Seminoma was observed in 29 participants and non-seminomatous germ cell tumor (NSGCT) in 10 participants.

Results

Nineteen patients were followed up with a high orchidectomy alone. After orchidectomy, one patient received prophylactic radiotherapy to the retroperitoneal lymph node, and three were followed by chemotherapy (BEP (bleomycin, etoposide, and platinum); BLM, VP-19/CDDP). Fifteen patients underwent retroperitoneal lymph node dissection (RPLND) after BEP. One patient received high-dose chemotherapy with peripheral blood stem cell transplantation (PBSCT), followed by RPLND, and then received additional BEP.

Twenty-six patients had azoospermia, and 13 patients were oligoasthenozoospermic. Sperm retrieval surgery was attempted in 18 patients with azoospermia. Thirteen patients underwent microscopic testicular sperm extraction (micro-TESE), two received onco-TESE, two received microscopic epididymal sperm aspiration (MESA), and one received retrograde vasal sperm aspiration (ReVSA) due to reduced efficacy of medications for ejaculatory dysfunction (EJD) caused by RPLND. Motile sperm recovery was obtained through micro-TESE (5/13), onco-TESE (2/2), MESA (2/2), and ReVSA (1/1). Eleven healthy deliveries were observed following intracytoplasmic sperm injection (ICSI) using surgically retrieved sperm.

Conclusions

Testicular cancer survivors, even those with azoospermia, can achieve fatherhood through ICSI after precise evaluation and appropriate sperm retrieval surgery.

## Introduction

Advances in surgery, radiotherapy, and chemotherapy have markedly improved the prognosis of cancer patients; however, infertility remains a significant concern among adolescents and young adults (AYAs; defined as between 15 and 39 years old). Although the incidence of testicular cancer is rare, it is the most common solid cancer among men in the AYA age group. Approximately 20,000 new cancer patients per year are in the AYA generation, thus accounting for 2.5% of all patients with cancer in Japan [1]. Cancer therapies can cause irreversible impairment of spermatogenesis. In testicular cancer patients, sperm quality is often compromised even at diagnosis [2,3], and treatment typically results in the loss of one testis, raising further concerns about future fertility. While cryopreservation of ejaculated sperm prior to treatment is recommended, it is still underutilized in clinical practice. In contrast, the use of cryopreserved ejaculated sperm for infertility treatment actually occurs in only 10% of patients [4]. In this article, we retrospectively evaluated the fertility status and treatment of testicular cancer survivors at our male infertility clinic.

This paper was presented at the poster and discussion session of the 41st Annual Meeting of the European Society of Human Reproduction and Embryology (ESHRE), held in Paris, France, from June 29 to July 2, 2025.

## Materials and methods

Study design, size, and duration

From 2004 through 2023, 3,361 male infertility patients were treated, 117 (3.5%) of whom had a history of cancer treatment. Thirty-nine patients diagnosed with testicular cancer were evaluated for cancer treatments, semen findings, and infertility treatments.

Testicular cancer type and treatment

The type of testicular cancer was seminoma in 29 patients, whereas it was non-seminomatous germ cell tumor (NSGCT) in 10 patients.

Nineteen patients with seminoma diagnosed as pT1N0M0 were followed up with a high orchidectomy alone, and one patient diagnosed as pT1N0M0 received prophylactic radiotherapy to the retroperitoneal lymph node. Patients diagnosed as pT1N2M0 (n = 1) and pT2N1M0 (n = 2) received BEP (bleomycin, etoposide, and platinum). Seminoma patients with pT2N0M0 (n = 1), pT2N1M0 (n = 2), and pT3N1M0 (n = 3) received BEP followed by retroperitoneal lymph node dissection (RPLND).

Patients diagnosed as having NSGCT pT2N0M0 (n = 1; embryonal/yolk sac/immature teratoma), pT2N2M0 (n = 1; embryonal/yolk sac/immature teratoma), pT3N0M0 (n = 1; embryonal), pT3N1M0 (n = 2; embryonal and embryonal/yolk sac/immature teratoma), pT3N1M1a (n = 1; seminoma/embryonal), pT3N1M1b (n = 2; embryonal/yolk sac and embryonal/yolk sac/immature teratoma), and pT3N2M1a (n = 1; embryonal/yolk sac) received BEP followed by RPLND. One NSGCT pT3N2M1b (embryonal) patient received high-dose chemotherapy assisted with peripheral blood stem cell transplantation (PBSCT), followed by RPLND, and then received two additional courses of BEP.

Sperm retrieval surgery

Sperm retrieval surgery was attempted in 18 patients with azoospermia. Thirteen patients underwent microscopic testicular sperm extraction (micro-TESE), two received onco-TESE, two received microscopic epididymal sperm aspiration (MESA), and one received retrograde vasal sperm aspiration (ReVSA). Onco-TESE was defined as a micro-TESE procedure performed at a sufficient distance from the tumor under ex vivo conditions.

Statement of ethics

The protocol for this research project, including its use of human subjects, was approved by a suitably constituted Ethical Committee of Kyoritsu General Hospital, Nagoya, Japan (approval number: 2024-12, dated March 3, 2025).

## Results

Patient characteristics are shown in Table 1. The median patient age was 36 years (interquartile range: 31-41), and the median spousal age was 33 years (30-36). Testicular volume was judged by a punched-out orchidometer. The median testicular volume was 12 mL (8-19) in the right testis and 14 mL (9-18) in the left. The median levels of luteinizing hormone (LH), follicle-stimulating hormone (FSH), and testosterone were 6.20 mIU/mL (4.15-10.80), 17.23 mIU/mL (8.68-23.05), and 3.87 ng/mL (3.43-5.28), respectively. Serum zinc concentration and body mass index were 76 (69-79) and 22.6 (20.8-24.8), respectively.

**Table 1:** Patient characteristics LH, luteinizing hormone; FSH, follicle-stimulating hormone

Parameter	Interquartile range
Age (y)	36 (31, 41)
Spouse age (y)	33 (30, 36)
Duration of infertility (y)	3.0 (2.0, 5.0)
Duration from termination of cancer treatment (y)	7.0 (2.3, 14.3)
Testicular size right/left (mL)	12 (8, 19)/14 (9, 18)
LH (mIU/mL)	6.20 (4.15, 10.8)
FSH (mIU/mL)	17.23 (8.68, 23.05)
Testosterone (ng/mL)	3.87 (3.43, 5.28)
Zinc (μg/dL)	76 (69, 79)
Body mass index (kg/m^2^)	22.6 (20.8, 24.8)

Semen analysis

Twenty-six patients had azoospermia, whereas 13 were oligoasthenozoospermic; mean sperm count and motility were 5.0 × 10^6^/mL (1.6-17.0) and 26% (14-75), respectively. The relationship between cancer stage and treatment is shown in Tables 2-3.

**Table 2:** Cancer stage and treatment (sperm present) NSGCT, non-seminomatous germ cell tumor; BEP, bleomycin/etoposide/cisplatin; RPLND, retroperitoneal lymph node dissection

Cancer type and stage	Treatment following orchidectomy
Seminoma pT1N0M0 (n = 8)	Orchidectomy alone
Seminoma pT2N1M0 (n = 2)	BEP
NSGCT1 pT2N0M0 (n = 1)	BEP/RPLND
NSGCT2 pT3N0M0 (n = 1)	BEP/RPLND
NSGCT2 pT3N1M0 (n = 1)	BEP/RPLND
NSGCT, NSGCT1: embryonal/yolk sac/immature teratoma, NSGCT2: embryonal	BEP/RPLND

**Table 3:** Cancer stage and treatment (no sperm) RPLND, retroperitoneal lymph node dissection; PBSCT, peripheral blood stem cell transplantation; NSGCT, non-seminomatous germ cell tumor; BEP, bleomycin/etoposide/cisplatin

Cancer type and stage	Treatment following orchidectomy
Seminoma pT1N0M0 (n = 11)	Orchidectomy alone
Seminoma pT1N0M0 (n = 1)	Radiotherapy
Seminoma pT1N2M0 (n = 1)	BEP
Seminoma pT2N0M0 (n = 1)	BEP/RPLND
Seminoma pT2N1Mo (n = 2)	
Seminoma pT3N1M0 (n = 3)	
NSGCT1 pT2N2M0 (n = 1)	
NSGCT1 pT3N2M0 (n = 1)	
NSGCT3 pT3N1M1a (n = 1)	
NSGCT1 pT3N1M1b (n = 1)	
NSGCT4 pT3N1M1b (n = 1)	
NSGCT4 pT3N2M1a (n = 1)	
NSGCT2 pT3N2M1b (n = 1)	High-dose chemotherapy (+PBSCT)/RPLND/BEP
NSGCT1: embryonal/yolk sac/immature teratoma, NSGCT2: embryonal, NSGCT3: seminoma/embryonal, NSGCT4: embryonal/yolk sac	High dose chemotherapy: CBDCA/etoposide/ifosfamide, PBSCT

Sperm retrieval surgery

In 18 patients who underwent sperm retrieval, motile sperm recovery was obtained through micro-TESE (5/13), onco-TESE (2/2), MESA (2/2), and ReVSA (1/1). Details of age, FSH value, cancer type and stage, treatment, and successful sperm retrieval are shown in Table 4.

**Table 4:** Successful sperm retrieval FSH, follicle-stimulating hormone; MESA, microscopic epididymal sperm aspiration; TESE, testicular sperm extraction; RPLND, retroperitoneal lymph node dissection; BEP, bleomycin/etoposide/cisplatin; ReVSA, retrograde vasal sperm aspiration; NSGCT, non-seminomatous germ cell tumor

Age	FSH value (mIU/mL)	Cancer type and stage	Treatment following orchidectomy	Sperm retrieval
41	6.4	Seminoma pT1N0M0	Orchidectomy alone	MESA
47	4.9	Seminoma pT1N0M0	Radiotherapy	MESA
31	7.3	Seminoma pT1N0M0	Orchidectomy alone	Onco-TESE
44	17.3	Seminoma pT1N0M0		Micro-TESE
46	19.4	Seminoma pT2N0M0	BEP/RPLND	Micro-TESE
37	16	NSGCT1 pT2N0M0		Micro-TESE
29	9.1	Seminoma pT3N1M0		Onco-TESE
27	17.2	NSGCT1 pT3N1M1b		Micro-TESE
29	9	NSGCT1 pT3N1M1b		Micro-TESE
36	8.4	NSGCT2 pT3N2M1b	High-dose chemotherapy/RPLND/BEP	ReVSA

Two patients with seminoma pT1N0M0 showed normal FSH values (6.4 and 4.9 mIU/mL). As dilated epididymal tubules were observed under an operative microscope, MESA was employed. These patients had epididymal obstruction due to unexplained causes, rather than impaired spermatogenesis caused by testicular cancer. 

Onco-TESE was performed on two seminoma patients. One case was pT3N1M0, and the contralateral testis was not clear on palpation or imaging. Another case, with a contralateral testis orchiectomized because of pT1N0M0 seminoma three years prior, also underwent onco-TESE. Both participants showed successful motile sperm recovery. One patient with NSGCT pT3N1M1b, treated with high-dose chemotherapy supported by PBSCT and an additional two courses of BEP after RPLND, showed ejaculatory dysfunction (EJD). Since he could not ejaculate after oral medication, ReVSA was employed, and motile sperm were recovered. Successful motile sperm retrieval was obtained through micro-TESE in seminoma pT1N0M0, seminoma pT2N0M0, NSGCT pT2N2M0, and two patients with NSGCT pT3N1M1b. Their mean FSH value was 17.2 mIU/mL (16.0-17.3).

Intracytoplasmic sperm injection (ICSI) results

One healthy boy and seven girls were born, and three boys were delivered through subsequent embryo transfer. Successful sperm retrieval and deliveries are shown in Table 5. 

**Table 5:** Successful sperm retrieval and delivery TESE, testicular sperm extraction; NSGCT, non-seminomatous germ cell tumor; MESA, microscopic epididymal sperm aspiration

Cancer type and stage	Sperm retrieval	Delivery
Seminoma pT1N0M0	MESA	Healthy boy
Seminoma pT1N0M0	Onco-TESE	Healthy girl
Seminoma pT1N0M0	Micro-TESE	Healthy girl
Seminoma pT2N0M0	Micro-TESE	Healthy girl, and healthy boy by subsequent embryo transfer
NSGCT1 pT2N2M0	Micro-TESE	Healthy girl, and healthy boy by subsequent embryo transfer
Seminoma pT3N1M0	Onco-TESE	Healthy girl, and healthy boy by subsequent embryo transfer
NSGCT1 pT3N1M1b	Micro-TESE	Healthy girl
NSGCT1 pT3N1M1b	Micro-TESE	Healthy girl

## Discussion

With advances in cancer treatment, the number of cancer survivors continues to rise. Fertility is a particularly important issue for AYA generations who wish to build families. Although relatively rare, testicular cancer is the most common solid malignancy in men of reproductive age. It typically develops at a young age, and both the disease and its treatments can impair spermatogenesis, hormone production, and sperm transport. Many patients already present with abnormal semen parameters at diagnosis, and the loss of a testis following treatment further compounds concerns regarding future fertility. BEP is considered the standard treatment for advanced testicular cancer. In general, the testicular toxicity of cisplatin, the main drug in BEP, is considered moderate. Levine et al. have reported an increased risk of testicular toxicity when cisplatin is administered at doses greater than 500 mg/m² [4]. In a prospective study, Pont and Albrecht reported that a cumulative cisplatin dose <400 mg/m² is unlikely to cause irreversible damage to fertility [5]. Since BEP is usually administered in three courses [6], the total cisplatin dose would be 300 mg/m², which is not associated with a high risk of toxicity. 

In a retrospective study, spermatozoa were extracted by TESE in 15 (65.2%) of 23 post-chemotherapy azoospermic patients [7]. The predominant histopathology patterns observed were Sertoli cells only (47.8%), hypospermatogenesis (30.4%), mixed (17.4%), and late maturation arrest (4.3%). On the other hand, Suzuki et al. reported that, among 45 testicular cancer patients treated with BEP, spermatogenesis was found in 44 [8]. Although the recovery of spermatogenesis was delayed according to the number of BEP cycles administered, the patients' age and semen parameters before chemotherapy were not predictive factors for the recovery of spermatogenesis. Shin et al. reported that the live birth rate was 27% in post-chemotherapy azoospermia, including hematopoietic cancer [9]. They also observed no significant differences in sperm retrieval rate, clinical pregnancy, or live birth rate between fathers with testicular cancer and hematopoietic cancer, and there were no predictive variables associated with successful sperm retrieval. However, irreversible damage may occur when anticancer drugs are used in high doses. In contrast, studies exploring additional BEP regimen adjustments to further decrease toxicity have shown that the lower threshold of efficacy has been reached, thus potentially compromising the efficacy of the chemotherapy [6]. Therefore, there is a dilemma between weighing the effectiveness of chemotherapy and the testicular damage caused by adverse effects.

In general, cancer survivors frequently have diminished testicular function after cancer treatment, and most cases of azoospermia may be considered non-obstructive azoospermia (NOA). Currently, micro-TESE is considered the standard surgery for obtaining motile sperm in NOA patients. We have previously reported that post-chemotherapy cancer survivors with NOA can achieve fatherhood through micro-TESE with ICSI [10]. Generally, advanced cancer requires more intensive treatment, resulting in a negative impact on testicular function. Although the number of cases was small, this was not necessarily the case in this study. We concluded that there does appear to be less correlation between the intensity of cancer treatment and semen findings [11]. In three cases in this study, TESE, which can cause testicular damage, should have been avoided. In two cases of seminoma pT1N0M0 diagnosed as having obstructive azoospermia, motile sperm were retrieved following MESA. Motile sperm were recovered using ReVSA [12] in an advanced NSGCT case (embryonal carcinoma; pT3N2M1b, FSH = 8.4 mIU/mL), who received high-dose chemotherapy (CBDCA/IFM) with PBSCT support and RPLND, followed by two courses of BEP. On the other hand, in 20 cases diagnosed as seminoma pT1N0M0 treated with orchidectomy alone, eight cases had motile sperm in their ejaculate, while 12 cases were azoospermic. In contrast, five patients with oligoasthenozoospermia had advanced testicular cancer diagnosed as seminoma pT2N1M0, NSGCT (embryonal/yolk sac/immature teratoma) pT2N0M0, NSGCT (embryonal) pT3N0M0, and NSGCT (embryonal) pT3N1M0. Cirigliano et al. reported that successful sperm recovery was obtained in 33.3% of cases through onco-TESE [13]. In our series, motile spermatozoa were extracted by onco-TESE in patients with pT3N1M0 seminoma with a single testicle and pT1N0M0 seminoma after contralateral orchidectomy two years previously. Thus, there may be individual variability rather than a strict correlation with cancer stage and subsequent treatment.

We previously reported that even in cases of cryptozoospermia with elevated FSH (suggesting testicular dysfunction), subsequent ICSI results were good [14]. If motile sperm can be obtained, ICSI can achieve a normal fertilization rate of 70.4% and a live birth rate of 73.8% per case. Repeated semen analyses are necessary for testicular cancer survivors, and, if motile sperm are present in the ejaculate, sperm cryopreservation should be considered.

Another important issue for testicular cancer survivors is EJD, commonly caused by RPLND. Even with nerve-sparing techniques, RPLND is associated with EJD in approximately 50%-60% of cases [15,16]. Two patients (NSGCT1; embryonal/yolk sac/immature teratoma, pT2N0M0, and NSGCT2; embryonal, pT3N0M0) developed EJD but regained the ability to ejaculate following oral administration of amoxapine (50 mg). Their semen analyses revealed sperm concentrations of 37 × 10^6^/mL and 6.5 × 10^6^/mL, with motility rates of 17% and 7%. However, in September 2022, the Ministry of Health, Labor, and Welfare of Japan reported the detection of N-nitrosoamoxapine, a potentially carcinogenic impurity, in amoxapine [17]. As a result, its supply was discontinued, and the drug is no longer available in Japan. Imipramine is currently used as an alternative, but its efficacy appears to be inferior to that of amoxapine [18]. Surgical options may be considered for drug-resistant EJD. In such patients, TESE is often performed for technical simplicity, even when testicular function is normal. However, recent reports indicate that even unilateral simple-TESE can increase the risk of postoperative hypogonadism [19,20]. To avoid unnecessary testicular damage, especially in testicular cancer survivors who already have a single testis, alternative strategies should be prioritized. We have previously shown that, in spinal cord-injured patients with normal FSH levels, motile sperm can be retrieved by ReVSA without testicular injury, and successful ICSI with such sperm has resulted in live birth [12].

In this study, TESE was avoided in three testicular cancer survivors. Although this number is small, it is important that motile sperm can be recovered without unnecessarily damaging the testicles. It is generally believed that an advanced cancer stage is associated with deteriorated testicular function; however, this may not necessarily be the case. TESE, employed as a sperm retrieval surgery, can cause damage to testicular function.

Limitations and reasons for caution

Although patients who presented with azoospermia were informed of the need for sperm retrieval surgery, several participants did not choose to undergo the operation. Because of a lack of information about pregnancy among participants with oligoasthenozoospermia, we cannot draw conclusions regarding their fertility status.

Wider implications of the findings

TESE is not always indicated for testicular cancer survivors with a single testicle. Surgeons should carefully determine the most appropriate sperm retrieval technique, as TESE - which can cause damage to the testis - should be used judiciously.

## Conclusions

Testicular cancer survivors are left with a single testicle following treatment. The preservation of spermatogenesis in these patients depends largely on individual potential, regardless of whether chemotherapy has been administered. It may be possible to retrieve motile sperm without TESE. Surgeons should carefully determine the most appropriate sperm retrieval technique to avoid unnecessary testicular damage.

Even in cases of azoospermia, fatherhood may still be achieved through accurate diagnosis, carefully selected sperm retrieval procedures that minimize postoperative testicular damage, and the use of ICSI.
